# Asymmetry of inter-joint coordination patterns and variability between dominant and non-dominant legs during landing in young healthy adults: a cross-sectional study

**DOI:** 10.1186/s13102-026-01949-6

**Published:** 2026-08-13

**Authors:** Xiaomei Hu, Shihan Chen, Cheuk Yin Ho, Xian Song, Yijian Yang

**Affiliations:** 1 Department of Sports Science and Physical Education, The Chinese University of Hong Kong, New Territories, Hong Kong China; 2 Department of Sports Science, Zhejiang University, Hangzhou, 310058 China

**Keywords:** lower limb coordination, Motor control, Side-to-side difference

## Abstract

**Background:**

Landing movement usually challenges motor control and causes lower limb injuries. Although asymmetries in landing biomechanics between the dominant and the non-dominant sides have been observed, the side-to-side differences in inter-joint coordination patterns and variability during landing are not fully understood.

**Methods:**

This study included 41 healthy young adults (21 females/20 males, age: 26.1 ± 5.5 years) who performed single-leg drop landings with inter-joint coordination patterns and variability being assessed. Modified vector coding was applied to calculate the coordination patterns and variability based on hip-knee sagittal, knee-ankle sagittal, knee sagittal-ankle frontal, and knee-ankle frontal coupling pairs. The coordination patterns were classified into in-phase with proximal, in-phase with distal, anti-phase with proximal and anti-phase with distal dominance. Paired-sample t-test was used to compare the limb differences.

**Results:**

Compared with the non-dominant side, the dominant side presented lower coordination variability (24.78 ± 7.37˚ vs. 27.47 ± 6.70˚, *p* = 0.044) in hip-knee sagittal coupling pair. The non-dominant side displayed higher frequency of anti-phase with distal dominance pattern in knee sagittal-ankle frontal coupling pair (27.25 ± 5.99% vs. 23.89 ± 5.13%, *p* = 0.020) when compared with the dominant side.

**Conclusion:**

Our results suggested that the dominant side tends to display more stable hip-knee sagittal coupling on the dominant side and ankle dominance modulation in knee sagittal-ankle frontal coupling on the non-dominant side. These distinct coordination profiles align with documented clinical trends reporting a higher prevalence of knee-related pathologies on the dominant side and an increased presence of ankle instability on the non-dominant side.

## Background

Landing is a functional movement during sports and daily activities, which challenges motor control and increases the risk of injury, particularly at knee and ankle joints [1–3]. Epidemiological survey reported that landing is one of the most common causes of injury, accounting for 32% of non-contact anterior cruciate ligament (ACL) injuries in football players and 45% of ankle injuries in basketball players [4, 5]. The loading exerted on lower extremity could alter motor patterns and increase joint coordination variability after landing, which may contribute to injuries [6]. Biomechanical analysis is a potential and valuable tool for capturing body movement and identifying motor patterns. Although biomechanical metrics of landing, like joint angle, angular velocity and moment, are widely used to screen injury risks and monitor injury recovery [7, 8], coordination patterns and variability can quantify the motor strategies and the flexibility of motor systems, which may provide insightful information to infer the inter-joint relationships associated with injuries.

Previous studies have shown evidence on how landing influences motor control and increases the risk of injuries [3, 9]. For instance, sagittal and frontal control of the knee joint, including reduced knee flexion angle and angular velocity, increased knee flexion moment, and increased knee abduction angle and moment, were found to be associated with ACL injury [3, 10, 11]. Some studies also observed that suboptimal ankle frontal and sagittal movement, such as increased inversion angle and angular velocity, and excessive ankle dorsiflexion, could lead to lateral ankle sprains [12, 13]. Previous study have suggested that altered motor regulations may be the reasons that induce the occurrence of knee and ankle injuries during landing [14]. Additionally, asymmetry between two legs might also be an important cause, since side-to-side differences have been suggested to be related to injuries and asymmetry has also been seen as a factor that influence the return to play in athletes [15, 16].

Asymmetry or laterality is mainly manifested in the symmetry of posture during movement and the preference for one side of the limbs during voluntary activities [14], which has been extensively investigated in terms of neurophysiology and motor control. Several studies have reported that there are differences in injury rates between the dominant and non-dominant sides in lower limb injuries among various sports activities [15, 17–19]. Matthew et al. [19] found there were 1.6 times more risk of injury to the dominant side in soccer players and Brophy et al. [18] reported that 74.1% of male soccer players experienced ACL injuries at the dominant side, owing to higher knee abduction angle and moments. The non-dominant side may exhibit a more effective landing strategy, with better shock absorption and a more balanced distribution of joint torque compared with the dominant side during landing in volleyball female players, which may be linked to higher ACL rate in dominant side [17]. Although the side-to-side differences were commonly reported in athletes, the asymmetric characteristics in healthy population, especially in general population, is still underexplored. Since they may be at higher risk of injury during sports due to insufficient training of the neuromuscular system when compared with athletes.

Although previous research have shown that biomechanical properties of individual joints during landing are not symmetrical, motor control plays an independent role to achieve stability by coordinating inter-joint motions during landing appropriately [20]. To quantify such inter-joint coordination patterns and variability, modified vector coding is applicable through calculating the coupling angles between two joints [21]. Based on this method, the four types of coordination patterns can elucidate the movement relationship between two joints [21]. In addition, the coordination variability can provide additional insights into how motor system adapts to the movement, reflecting stable or unstable motor regulation. Several studies have used modified vector coding technique to quantify the coordination variability and explore the coordination pattern characteristics in ankle or knee injuries [22–24]. However, whether there are side-to-side differences regarding coordination patterns and variability between dominant and non-dominant sides in healthy adults remains unclear. Therefore, this study aimed to investigate the coordination patterns and variability between the dominant and non-dominant sides during landing in healthy adults. Moreover, traditional kinematic variables, joint angles, were included to enhance comparability with previous studies. We hypothesized that healthy individuals would exhibit coordination patterns and variability asymmetries, with the dominant side demonstrating less variability (i.e., stable).

## Methods

### Participants

In this cross-sectional study, the sample size was calculated using G*Power software version 3.1.9.7 by assuming an alpha level of 0.05, power of 0.8, effect size of 0.5 based on the empirical assumption, resulting in 34 participants. Finally, 41 healthy and physically active participants (21 females and 20 males, age: 26.1 ± 5.5 years, height: 169.9 ± 9.2 cm, mass: 62.7 ± 8.5 kg, body mass index: 21.7 ± 2.4 kg/m²) were recruited from the local community and universities. Thirty-six were right-dominant side and five were left-dominant side. The limb dominance was determined as the preferred leg to kick a ball based on previous method [25]. The participants were asked: If I gave you a ball, which leg would you prefer to kick it. The inclusion criteria included: (1) age between 18 and 40 years old; (2) non-elite athletes (never participated in international competitions, national or professional competitions) [26]; and (3) no history of lower limb injury, or diagnosed neurological or vestibular disorders. Exclusion criteria include: (1) current pain or injury affecting lower limb function; and (2) previous lower limb surgery. This study was approved by the Survey and Behavioural Research Ethics of the Chinese University of Hong Kong, Faculty of Education (SBRE-24-0645) and conducted in accordance with the ethical principles outlined in the Declaration of Helsinki. All participants provided the written inform consent.

### Data collection

All participants were required to complete the drop-landing test, twenty reflective markers (9-mm diameter, Table 1) were placed to capture the lower limb movement during landing using 12-camera motion capture system (Vicon Bonita 10, Oxford, UK) sampling at 100 Hz. A static trial was recorded while maintaining a “T” position (with feet shoulder-width apart and arms to the side parallel with the ground). Before formal tests, participants were given a five-minute warm-up and up to 10 landing practices. Then, they were required to land from a 45-cm-high platform barefoot with one leg on the force plate and maintain balance for at least three seconds [27]. The contralateral side was not permitted to touch the ground. Sufficient rests were allowed between trials and the order was randomized. Five successful trials on each side were used for analysis. A standardized drop height of 45 cm was selected because it represents a demanding load sufficient to challenge lower-extremity neuromuscular control, and it aligns with established protocols in landing biomechanics investigation [28].

**Table 1:** Marker placement

Segment	Markers placed on the segment (one on each side, total = 20)
Pelvis	anterior superior iliac spine, posterior superior iliac spine
Thigh	greater trochanter, lateral epicondyle, medial epicondyle
Shank	lateral malleolus, medial malleolus
Foot	calcaneus, head of the first metatarsal, head of the fifth metatarsal

### Data processing

A rigid body of lower extremity (pelvis, thigh, shank and foot) was modeled using the static trial with right-handed coordinate system and was applied to all landing trials. All kinematic and kinetic data were analyzed in Visual 3D software version 2024. 11. 2 (C-Motion, Inc., Germantown, MD, USA). The marker trajectories and force data were filtered using a fourth-order, zero-lag Butterworth low-pass filter with cut-offs at six and 50 Hz, respectively [29]. Joint angles were calculated from initial contact, determined by a 10-N ground reaction force threshold, to maximal knee flexion [29].

Then, the joint angle were used to calculate the coupling angles (γ) between two joints using the modified vector coding technique [21]. Since knee and ankle injuries are usually caused by suboptimal sagittal and frontal movements, the following coupling pairs were analyzed: hip sagittal-knee sagittal, knee sagittal-ankle sagittal, knee sagittal-ankle frontal, knee frontal-ankle frontal coupling pairs [23, 24, 30]. The coupling angles were then normalized to 101 points using linear interpolation (i.e., representing 0-100% of the landing phase) for each landing trial and averaged for the five trials for further analysis. The coordination variability is the standard deviation of coupling angles at each normalized time point during landing; based on specific ranges of coupling angles, the coordination pattern can be classified into in-phase with proximal dominance, in-phase with distal dominance, anti-phase with proximal dominance and anti-phase with distal dominance (Table 2) [21].

**Table 2:** Explanation of coordination pattern

Category	Range of coupling angle	Kinematics interpretation
In-phase with proximal dominance	0˚≤ γ ≤ 45˚, 180˚ < γ ≤ 225˚	Two joints move on the same direction and proximal joint moves more than distal joint.
In-phase with distal dominance	45˚ < γ ≤ 90˚, 225˚ < γ ≤ 270˚	Two joints move on the same direction and distal joint moves more than proximal joint.
Anti-phase with proximal dominance	135˚ < γ ≤ 180˚, 315˚ < γ < 360˚	Two joints move on the opposite direction and proximal joint moves more than distal joint.
Anti-phase with distal dominance	90˚ < γ ≤ 135˚, 270˚ < γ ≤ 315˚	Two joints move on the opposite direction and distal joint moves more than proximal joint.

### Statistical analysis

Continuous data were expressed as mean and standard deviation. Significant level was set at alpha = 0.05. Normality was checked by the Shapiro-Wilk test. For non-normally distributed data, log transformation was used. Paired-sample t-tests were applied to compare the coordination patterns and variability between dominant and non-dominant sides across full sample in SPSS (Version 31.0, SPSS Inc., Chicago, IL). Considering multiple coupling pairs used, false discovery rate correction using the Benjamini-Hochberg procedure was used to control the Type I error [31]. Cohen’s d and 95% confidence intervals were reported as small (≥ 0.2), moderate (≥ 0.5) and large (≥ 0.8) effect size (ES) [32]. 1D-statistical parametric mapping (1D-SPM) of paired-sample t-test was conducted in MATLAB to analyze coupling angles and joint angles (i.e., hip, knee and ankle frontal and sagittal angles) during landing (www.spm1d.org). Since 1D-SPM has accounted for multiple comparisons across the continuum using random field theory and controlled the family-wise error rate over the entire waveform, therefore the false discovery rate correction was not used for SPM analysis [33].

## Results

**Fig. 1:**
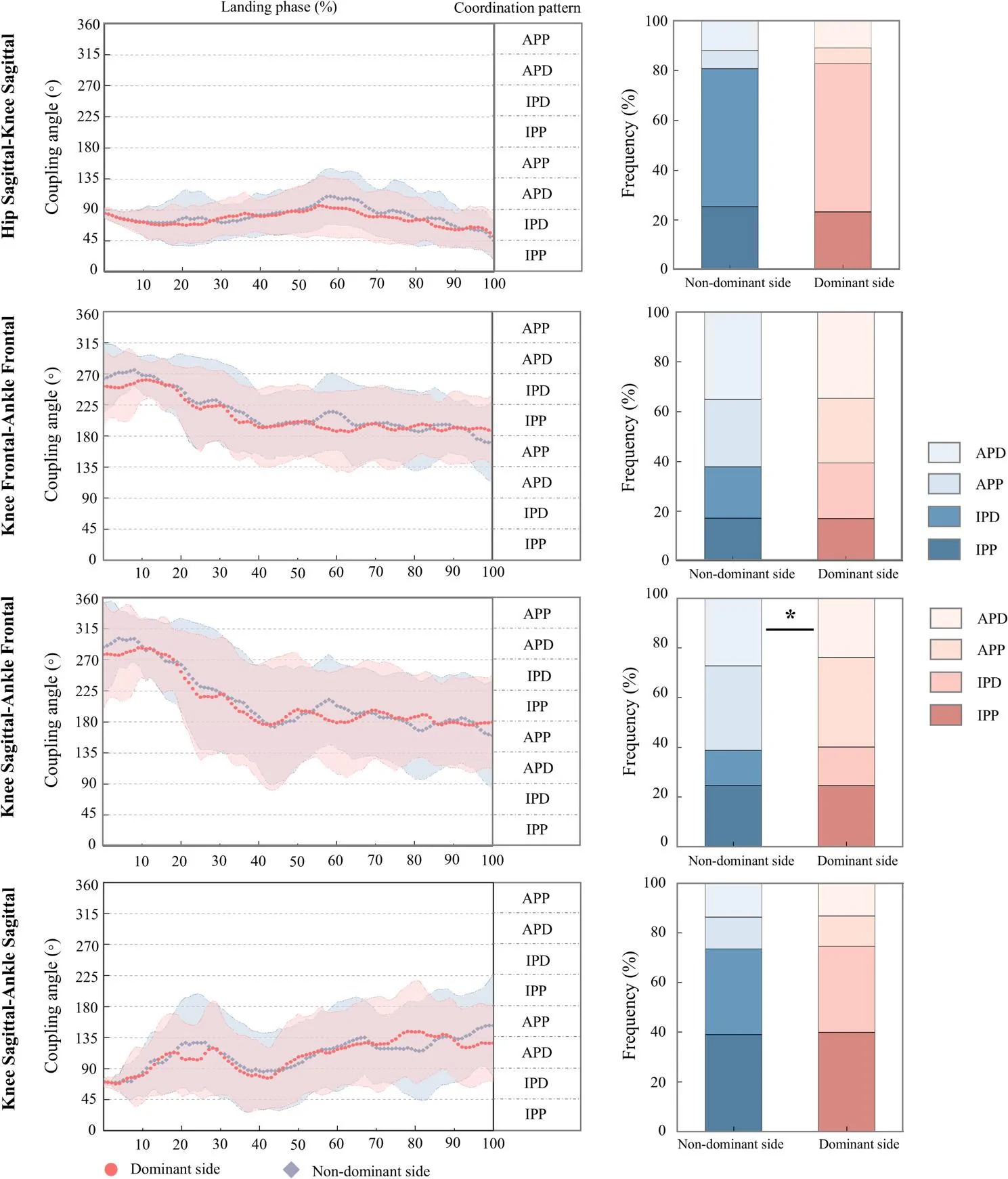
Coordination patterns and coupling angles of coupling pairs. APD: anti-phase with distal dominance, APP: anti-phase with proximal dominance, IPD: in-phase with distal dominance, IPP: in-phase with proximal dominance. *p < 0.05, significant between-side difference

For coordination variability (Table 3), paired-sample t-test showed a significant difference in the hip sagittal-knee sagittal coupling pair between dominant and non-dominant sides (*p* = 0.044, ES = 0.42) after multiple comparison correction. While for coordination pattern, significant differences were observed for knee sagittal-ankle frontal coupling pair of anti-phase with distal dominance pattern (*p* = 0.020, ES = 0.46, Fig. 1). No significant differences were found for coordination variability of other coupling pairs (*p* ≥ 0.812) and other coordination patterns (*p* ≥ 0.062). SPM analysis of coupling pattern did not reveal any significant differences between two sides during the impact phase of landing (*p* > 0.05).

**Table 3:** Coordination variability of coupling pairs

Coupling pair	Non-dominant	Dominant	*p* value	t value	ES (95% CI)
Hip sagittal-knee sagittal	27.47 ± 6.70˚	24.78 ± 7.37˚	**0.044**	2.681	0.42 (0.10, 0.74)
Knee sagittal-ankle sagittal	39.38 ± 6.11˚	39.49 ± 5.66˚	0.868	−0.167	−0.03 (− 0.33, 0.28)
Knee sagittal-ankle frontal	38.95 ± 4.38˚	39.61 ± 5.35˚	0.812	−0.839	−0.13 (− 0.44, 0.18)
Knee frontal-ankle frontal	46.52 ± 3.38˚	46.29 ± 4.11˚	0.980	0.341	0.05 (− 0.25, 0.36)

Bold text represents statistical significance

The *p* value was adjusted by false discovery rate correction using the Benjamini-Hochberg procedure; Effect size and 95% confidence intervals calculating by non-central t-distribution were extracted from IBM SPSS Version 31.0, the sign of the effect size indicates directionality, calculated consistently as non-dominant minus dominant side

*CI* Confidence interval, *ES* Effect size

**Fig. 2:**
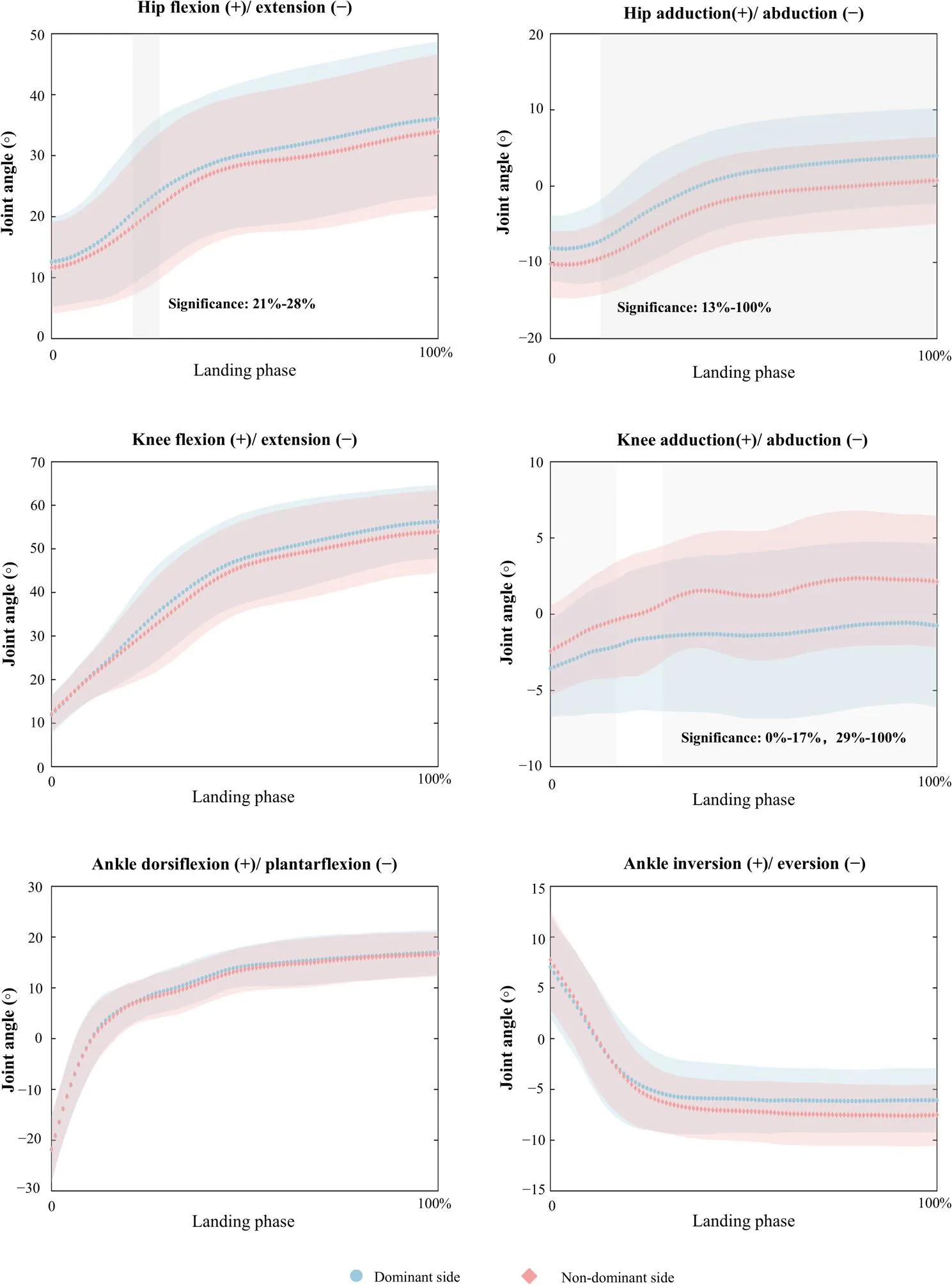
SPM analysis of joint angle during landing. Shade areas represent statistical significance (*p* < 0.05)

SPM analysis of joint angles (Fig. 2) revealed greater hip flexion during 21%-28% of landing phase (*p* = 0.047) on the dominant side. Additionally, greater knee abduction during 0–17% (*p* = 0.034) and 29%-100% (*p* < 0.001) of landing phase were also observed on the dominant side. Compare with the dominant side, the non-dominant side presented greater hip abduction during 13% to 100% of landing phase (*p* = 0.003).

## Discussion

The study compared the differences in coordination patterns and variability between the dominant and non-dominant sides after landing. We found that the dominant side exhibited stable, coupled movement strategy in the hip sagittal-knee sagittal coupling pair alongside greater knee abduction and hip adduction after landing. The non-dominant side displayed greater ankle regulation pattern in knee sagittal-ankle frontal coupling pair. These observations suggested that there were asymmetries between the dominant and non-dominant sides regarding the inter-joint coordination pattern and variability. The dominant limb may operate under a more rigid, stability-driven control strategy to manage high-intensity loads, whereas the non-dominant limb may employ a distal-regulation strategy to accommodate high post-landing variability. However, it remains entirely unknown how these specific coordinative adjustments translate into long-term potential injury risk.

Compared with the non-dominant side, the dominant side presented lower coordination variability in the hip sagittal-knee sagittal coupling pair, indicating more stable coordination between hip and knee sagittal movement during the impact phase of single-leg landing. The dominant side may display stronger motor control capabilities with long-term motor learning and functional optimization, thereby demonstrating greater efficiency in movement modulation [34]. Although Haaland et al. [35] suggested that the dominant hemisphere and limb are specialized for predictive control of intersegmental limb dynamics, this perspective is best viewed as a theoretical reference parallel to our findings. For landing, this precise control is likely reinforced by long-term behavioral limb preference and task-specific learning, rather than being merely a direct manifestation of hemispheric functional specialization. While lower variability typically reflects a stable motor control, it also indicates a tightly regulated strategy to manage landing forces. This rigid motor pattern limits a participant’s ability to adapt to sudden perturbations [36]. Moreover, invariant coordination repetitively loads the exact same musculoskeletal tissues over multiple trials, potentially elevating overuse injury risk. We found greater knee abduction angle throughout almost the whole landing phase compared with non-dominant side, which may result in greater knee joint loading and may lead to increased knee-related injuries on the dominant side, especially for ACL injury [3].

Additionally, the non-dominant side displayed greater frequency of anti-phase of distal dominance in knee sagittal-ankle frontal coupling pair indicates that relatively more ankle frontal movement may be used to regulate posture on non-dominant side. Interestingly, this coordination difference occurred despite a lack of significant between-limb differences in discrete ankle inversion and eversion angles. This mismatch highlights that coordination analysis evaluates the relative, dynamic movement between two segments, which reflects motor flexibility and adaptability, rather than simple, discrete kinematic alterations [21]. While our dataset cannot directly confirm the underlying neural mechanisms, it is possible that proactive, feedforward regulation altered joint coordination to preemptively stabilize the ankle and mitigate injury risk without manifesting as overt changes in joint angles. This strategy may have been supported by the standardized landing instructions and familiarization practice provided to participants. Future studies incorporating electromyography are needed to verify these neuromuscular control strategies. Nonetheless, a tendency toward a higher range of motion in ankle frontal plane movement may elevate dynamic instability in the non-dominant ankle. This is particularly relevant given that previous studies have identified altered coordination variability in the knee sagittal-ankle frontal coupling pair among individuals with a history of ankle sprains [23, 37].

In summary, the asymmetries in coordination variability and patterns partially aligns with traditional kinematics metrics, providing valuable insights into understanding how leg dominance results in specific joint regulation and clarifying the inter-joint relationships during landing. However, this study has several limitations that should be acknowledged. First, in this study, we used 45-cm platform, the result may not be generalizable to different landing heights, as the platform height could potentially alter landing mechanics and postural regulation [38]. Second, we only included general population, while the elite athletes may display smaller coordination variability, especially for sports that require repetitive jumping and landing. Additionally, we determined the dominant side using kicking task, however the limb dominance is task-dependent and that the preferred kicking leg does not always dictate the preferred landing leg, which may influence the interpretation of potential functional asymmetries in current study. Finally, although both male and female participants were included, their physical activity levels may not fully matched and we did not consider the gender effect. Previous research has reported that gender may interact the leg dominance during landing [39]. Therefore, our findings should be generalized across both sexes with caution. Nevertheless, the findings are applicable to physically active young adults in general, and help understand limb asymmetries and explain the joint-specific regulation characteristics. Future studies may also consider muscles coordination features to comprehensively understand the motor control differences between dominant and non-dominant sides.

## Conclusion

This study observed distinct inter-joint coordination asymmetries between limbs after landing, characterized by more stable hip-knee sagittal coupling on the dominant side and ankle dominance modulation in knee sagittal-ankle frontal coupling on the non-dominant side. These characteristics align structurally with the distribution of joint-specific vulnerability across limbs documented in previous studies. These side-to-side differences may provide valuable insights for understanding asymmetric lower-limb loading, suggesting that monitoring the dominant knee, non-dominant ankle, and their coupling with neighboring joints is warranted. These observational findings suggest that practitioners may utilize joint coordination analysis to design task-specific training programs aimed at managing inter-limb asymmetries during landing.

## Data Availability

The datasets generated and/or analysed during the current study are available from the corresponding author on reasonable request.

## Abbreviations

ACL: Anterior cruciate ligament
ES: Effect size
SPM: Statistical parametric mapping
